# Misrecognition of facial expressions in delinquents

**DOI:** 10.1186/1753-2000-3-27

**Published:** 2009-09-18

**Authors:** Wataru Sato, Shota Uono, Naomi Matsuura, Motomi Toichi

**Affiliations:** 1 Department of Comparative Study of Cognitive Development (Funded by Benesse Corporation), Primate Research Institute, Kyoto University, Inuyama, Aichi 484-8506, Japan; 2 Department of Cognitive Psychology in Education, Faculty of Education, Kyoto University, Yoshida-honmachi, Sakyo-ku, Kyoto 606-8501, Japan; 3 Graduate School of Education, Tokyo University of Social Welfare, Higashi-Ikebukuro, Toshima-ku, Tokyo 170-8426, Japan; 4 Graduate School of Human Health Science, Faculty of Medicine, Kyoto University, Shogoin Kawara-cho, Sakyo-ku, Kyoto 606-8057, Japan

## Abstract

**Background:**

Previous reports have suggested impairment in facial expression recognition in delinquents, but controversy remains with respect to how such recognition is impaired. To address this issue, we investigated facial expression recognition in delinquents in detail.

**Methods:**

We tested 24 male adolescent/young adult delinquents incarcerated in correctional facilities. We compared their performances with those of 24 age- and gender-matched control participants. Using standard photographs of facial expressions illustrating six basic emotions, participants matched each emotional facial expression with an appropriate verbal label.

**Results:**

Delinquents were less accurate in the recognition of facial expressions that conveyed disgust than were control participants. The delinquents misrecognized the facial expressions of disgust as anger more frequently than did controls.

**Conclusion:**

These results suggest that one of the underpinnings of delinquency might be impaired recognition of emotional facial expressions, with a specific bias toward interpreting disgusted expressions as hostile angry expressions.

## Background

In recent years, increasing attention has been focused on the high rate of delinquency, which is a serious social problem in some countries [1]. To address this problem, it is important to clarify the psychological mechanisms underlying conduct problems in youths. Some clinical observations and questionnaire surveys have revealed deficits in emotional communication among children and adolescents with conduct problems (e.g., [2]).

One crucial component of emotional communication is the recognition of emotional facial expressions of other individuals. Facial expressions indicate moment-to-moment changes in inner emotional states [3] and/or communicative intentions [4]. People often use the information communicated by emotional facial expressions as cues for modulating social behaviors [5]. In particular, the recognition of others' facial expressions has been shown to modulate aggressive behaviors [6]. This finding suggests that there may be a relationship between facial expression recognition and conduct problems involving aggression.

Consistent with this notion, some previous studies have revealed that delinquents are impaired in their recognition of facial expressions of emotion [7-9]. However, the types of emotion they have difficulty recognizing have not been clearly identified. For example, McCown et al. [9] investigated the recognition of facial expressions of six basic emotions (anger, disgust, fear, happiness, sadness, and surprise; cf. [3]) among incarcerated juvenile delinquents. They found that, compared with control youths, juvenile delinquents were less accurate in the recognition of facial expressions of disgust, sadness, and surprise. On the other hand, Cadesky et al. [7] investigated the recognition of facial and vocal expressions of anger, fear, happiness, and sadness in children with conduct problems. They reported that these children were impaired in the recognition of fear, happiness, and sadness. In summary, although the previous studies have consistently indicated the impairment of facial expression recognition in delinquents, it remains unclear whether there is a specific pattern of impairment.

Cadesky et al. [7], in their subsequent analysis, examined the error patterns that suggested poor emotion recognition among delinquent participants. By conducting visual inspections of their data, they found that participants with conduct problems tended to mislabel other emotions as anger. Because they did not conduct statistical analyses, their conclusion should be regarded as tentative. This finding, however, seems to provide an important clue regarding how delinquents misperceive others' emotional expressions. Several researchers have reported a similar tendency among children with conduct problems to misperceive benign social situations as hostile [10-12].

In the present study, we investigated facial expression recognition in adolescent/young adult delinquents in greater detail than have previous studies, comparing delinquents with age- and gender-matched controls. We examined participants' recognition of facial expressions conveying the six basic emotions previously examined by McCown et al. [9] and conducted error analyses for each emotion. We predicted that delinquents would recognize facial expressions of some emotions less accurately than would control participants, with a bias toward the misinterpretation of emotions as anger. Given that some previous studies have reported cultural differences in expression recognition (e.g., [13]), we used facial-expression stimuli from two different cultures.

## Methods

### Participants

Twenty-four male adolescent/young adult delinquents (mean age ± *SD*, 18.3 ± 1.3 years) participated in this study. They were incarcerated in two correctional facilities in Japan, A (*n *= 13) and H (*n *= 11). Statistical data have suggested that Japan's rate of delinquency is comparable to those of some Western countries (e.g., France) [14]. In Japan, however, the proportion of delinquents who are incarcerated in correctional facilities is very low; in 2004, only 0.05% of delinquents who had been arrested were incarcerated in correctional facilities [1]. The fact that the participants of this study were in correctional facilities indicates that they had severe conduct problems. Results of the Japanese version of the Child Behavior Checklist (CBCL) [15,16] completed by their teachers have confirmed severe conduct problems in our participants (Table 1). We found no significant differences between the two facilities in subscale or total scores on the CBCL (*t *-test, *P*s > 0.1). The mean full-scale intelligence quotient (IQ) of these delinquents, measured by the revised Wechsler Adult Intelligence Scale (WAIS-R) or revised Wechsler Intelligence Scale for Children (WISC-R), was in the normal range (mean ± SD full-scale IQ = 85.1 ± 11.3; mean ± SD verbal IQ = 84.7 ± 10.9; mean ± SD performance IQ = 87.9 ± 11.6).

**Table 1:** Mean T-scores (with *SD*) for the Child Behavior Checklist among juvenile delinquents.

**Subscale**	**M**	**SD**
Social withdrawal	56.2	6.1
Somatic complaints	54.1	5.7
Anxiety/depression	58.6	7.2
Social problems	56.3	7.4
Thought problems	55.4	5.5
Attention problems	58.9	8.5
Delinquent behavior	70.3	9.9
Aggressive behavior	62.9	13.1
Internalizing behavior	55.5	9.4
Externalizing behavior	66.1	12.3
Total	66.9	8.9

Twenty-four age- and gender-matched participants (mean age ± *SD*, 17.4 ± 3.5 years; *t*-test, *t*(46) = 1.54, *P *> 0.1; all males) served as controls. They were recruited through advertisements and participated in the experiment as volunteers. Their IQs were also measured by the WAIS-R or WISC-R (mean ± SD full-scale IQ = 108.6 ± 18.3; mean ± SD verbal IQ = 113.1 ± 21.8; mean ± SD performance IQ = 101.4 ± 13.8). The IQs of control participants were significantly higher than were those of delinquent participants (t(46) = 5.62, *P *< 0.001).

All participants were born in Japan, and their first language was Japanese. All participants had normal or corrected-to-normal visual acuity. All participants gave informed consent to participate in this study, which was conducted in accordance with the ethical provisions of the institution and the Declaration of Helsinki. No candidate refused to participate in the experiment.

### Stimuli

A total of 48 photographs of facial expressions depicting six basic emotions (anger, disgust, fear, happiness, sadness, and surprise) were used as stimuli. Half of these pictures consisted of Caucasian models and the remaining half consisted of Japanese models. The pictures of Caucasian and Japanese models were chosen from the standard facial image sets of Ekman and Friesen [17] and Matsumoto and Ekman [18], respectively.

### Apparatus

The events were controlled by SuperLab Pro 2.0 (Cedrus), implemented on a laptop Windows computer (Inspiron 8000, Dell).

### Procedure

A label-matching paradigm used by a previous neuropsychological study [19] was employed to assess recognition of facial expressions. Pictures of people whose faces expressed various emotions were presented on the monitor one by one in a random order. Verbal labels identifying the six basic emotions were presented next to each photograph. Participants were asked to select the label that best described the emotion shown in each photograph. They were instructed to consider all six alternatives carefully before responding. No time limits were set, and no feedback was provided about performance during the test trials. Participants saw each emotional expression eight times, resulting in a total of 48 trials for each participant.

To confirm adequate understanding of the emotional labels, we interviewed participants before testing began, asking them to provide examples of situations that would elicit each of the emotions. All participants were able to give appropriate examples without difficulty. After this interview, participants completed five practice trials to become familiarized with the procedure.

### Data Analysis

The data were analyzed using SPSS 10.0J (SPSS Japan). The percentages of accurate responses were analyzed with a 2 (group) × 6 (facial emotion) × 2 (stimulus type) design. Full-scale IQ and age were included in the analysis as covariates. To appropriately process the violation of the sphericity assumption for the repeated-measures design, data in the levels of the within-subjects independent variables were viewed as separate dependent variables, and a multivariate analysis of covariance (MANCOVA) was conducted (cf. [20]). For significant interactions related to the group factor, follow-up multivariate analyses of variance (MANOVAs) were conducted with Bonferroni's correction; the α level was divided by the number of statistical tests performed (i.e., 6 for facial emotions). The adjusted P values were reported (cf. [21]). Similar follow-up analyses were also conducted for other significant main effects and interactions. For these multivariate analyses, Wilks' λ criterion was used. The percentages of erroneous responses were analyzed for facial emotions that showed a significant effect of group. The percentage of erroneous responses for each emotional label was calculated as the rate of erroneously selected labels in all trials for that facial expression. Based on our prediction, a t-test comparing groups was conducted for selections of the anger label. Because the aforementioned follow-up MANOVAs on the accuracy of recognition did not show any significant interactions between group and stimulus type, the factor of stimulus type was collapsed.

## Results

### Accuracy

**Figure 1:**
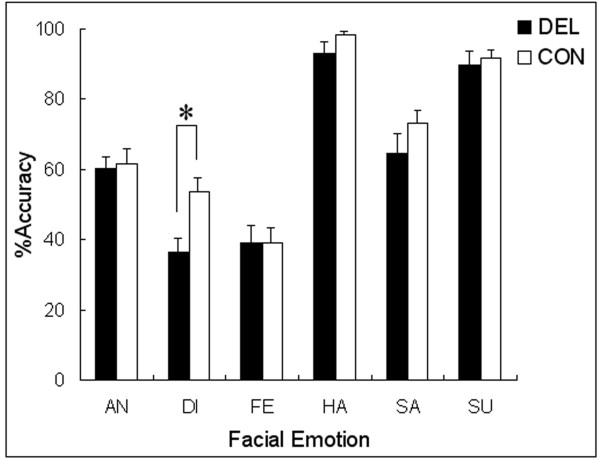
**Mean (with *SE*) percentages of accurate facial emotion recognition collapsed across stimulus types in delinquents (DEL) and controls (CON)**. An asterisk indicates a significant difference between groups (*P *< 0.05). AN = anger; DI = disgust; FE = fear; HA = happiness; SA = sadness; SU = surprise.

The MANCOVA for the percentages of accurate responses (Table 2; Figure 1) revealed a significant interaction of group × facial emotion × stimulus type (*F *[5, 40] = 3.65, *P *< 0.01), which was the only significant effect found with respect to the group factor (cf. main effect of group: *F *[1, 44] = 1.44; interaction of group × facial emotion: *F *[5, 40] = 0.50; interaction of group × stimulus type: *F *[1, 44] = 1.89; *P*s > 0.1). We also found a significant main effect of emotion (*F *[5, 40] = 4.40, *P *< 0.005), a significant interaction of emotion × stimulus type × IQ (*F *[5, 40] = 2.68, *P *< 0.05), and a significant interaction of emotion × stimulus type × age (*F *[5, 40] = 3.30, *P *< 0.05). Trends toward significance were found for the main effect of age (*F *[1, 44] = 3.64, *P *< 0.1) and the interaction of emotion × stimulus type (*F *[5, 40] = 2.15, *P *< 0.1). Other main effects or interactions were not significant (*P*s > 0.1).

**Table 2:** Mean (with *SE*) percentages of accurate facial emotion recognition.

			**Facial emotion**					
			
**Group**	**Stimulus type**		**AN**	**DI**	**FE**	**HA**	**SA**	**SU**
Delinquent	Caucasian	M	54.2	42.7	36.5	88.5	57.3	83.3
		SE	(5.4)	(5.5)	(6.4)	(6.0)	(6.5)	(6.0)
	Japanese	M	66.7	30.2	41.7	96.9	71.9	95.8
		SE	(4.4)	(6.4)	(5.8)	(1.7)	(6.8)	(2.5)
Control	Caucasian	M	60.4	65.6	46.9	97.9	74.0	94.8
		SE	(5.8)	(5.2)	(5.1)	(1.4)	(4.9)	(2.1)
	Japanese	M	62.5	41.7	31.3	99.0	71.9	88.5
		SE	(5.2)	(6.5)	(6.1)	(1.0)	(4.8)	(4.3)

AN = anger; DI = disgust; FE = fear; HA = happiness; SA = sadness; SU = surprise.

As follow-up analyses on the interaction of group × facial emotion × stimulus type, we conducted an analysis with the factors of group and stimulus type for each facial emotion using a MANOVA with Bonferroni's correction (α = 0.008). The results revealed that the main effect of group was significant for the facial expressions depicting disgust, indicating less accurate recognition in delinquents than in control participants (*F *[1, 46] = 8.93, adjusted *P *< 0.05). The main effect of stimulus type was also significant for expressions of disgust, indicating more accurate recognition in response to Caucasian than to Japanese faces (*F *[1, 46] = 8.96, adjusted *P *< 0.05). Other main effects or interactions were not significant (adjusted *P*s > 0.1).

Follow-up analyses were conducted for the main effect of facial emotion to clarify overall patterns of expression recognition. The Bonferroni-corrected (α = 0.003) MANOVAs showed the following significant differences (*F*s [1, 47] > 14.60, adjusted *P*s < 0.01): happy and surprised expressions were recognized with greater accuracy than were other expressions; sad and angry expressions were recognized with greater accuracy than were disgusted and fearful expressions.

To test whether additional factors influenced the recognition of facial expressions of disgust, follow-up analyses were conducted for the other significant three-way interactions. We conducted a two-way analysis with Bonferroni's correction (α = 0.008) for each facial emotion. For the interaction of facial emotion × stimulus type × IQ, the main effect of stimulus type and the interaction of stimulus type × IQ were significant for surprised expressions (*F*s [1, 46] = 15.27 and 14.44, respectively, adjusted *P*s < 0.01), and no other significant main effects or interactions were found (adjusted *P*s > 0.1). For the interaction of facial emotion × stimulus type × age, no significant main effects or interactions were found (adjusted *P*s > 0.1). In summary, factors other than group and stimulus type had no significant effect on the recognition of disgusted expressions.

### Error

**Figure 2:**
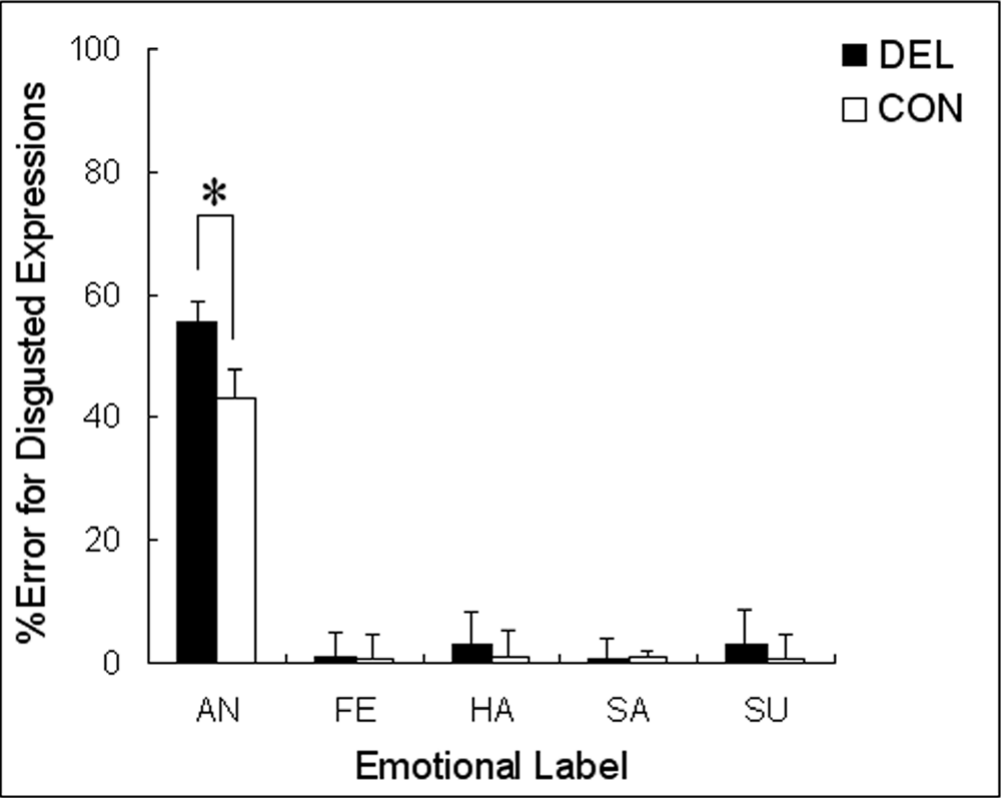
**Mean (with *SE*) percentages of errors for the recognition of disgusted facial expressions in delinquents (DEL) and controls (CON)**. An asterisk indicates a significant difference between groups (*P *< 0.05). AN = anger; FE = fear; HA = happiness; SA = sadness; SU = surprise.

The *t*-test showed that delinquents selected anger as the label to describe disgusted expressions more frequently than did control participants (Figure 2; *t *[46] = 2.30, *P *< 0.05).

## Discussion

Our results revealed that adolescent/young adult delinquent participants were less accurate in recognizing facial expressions of disgust than were control participants. The problems in the facial expression recognition among delinquents are consistent with findings of previous studies [7-9]. More specifically, the present results are consistent with a previous study in identifying impairment in the recognition of disgust [9]. Despite methodological differences among studies, such as differences in the cultural background of participants, the present study is compatible with previous studies in suggesting that delinquents have impaired ability to recognize emotional facial expressions.

Our results on errors revealed that delinquents had a tendency to misrecognize facial expressions of disgust as anger. Although this type of error was also prominent in control participants, which is plausible because angry and disgusted facial expressions are similar with respect to featural changes and both express negative emotional states [3], delinquents showed a much greater tendency than did control participants to exhibit this misrecognition. Although the difference in error rates between groups was not large (17.2%), such errors can provide valuable information regarding impairments in expression recognition (e.g., [19]). This error pattern is consistent with the suggestion by Cadesky et al. [7] that children with conduct problems tend to perceive other emotions as anger. The present results also agree with previous reports that participants with conduct problems misperceived social situations as hostile [10-12], although those studies did not focus on the recognition of facial expressions. Extending these previous findings, the present study provides the first clear evidence that delinquents have a bias toward the misrecognition of others' disgusted expressions as anger.

Delinquents' misperception of facial expressions of disgust as anger is important when we consider the social functions of these emotions. Although both angry and disgusted facial expressions induce negative emotional states in perceivers, angry expressions induce higher arousal than do disgusted expressions [22]. Furthermore, disgusted facial expressions suggest withdrawal motivation on the part of the sender, whereas angry expressions indicate approach motivation [23]. Specifically, angry facial expressions imply the occurrence of subsequent hostile behaviors [24]. These data suggest that the misrecognition of disgusted facial expressions as angry expressions might induce relatively more intense emotionally aroused states in the receiver, and might result in anticipation of relatively more dangerous behavior on the part of the sender than would accurate recognition. These misperceptions of facial expressions might therefore contribute to aggressive behaviors in delinquents.

The bias toward misrecognizing other emotions as anger is particularly significant because anger appears to play an important role in delinquency. It has been pointed out that children with conduct problems are quicker to become angry and their anger tends to be more intense [25]. Plattner et al. [26] confirmed that delinquents experienced higher state and trait negative emotions, including anger, than did control participants. A previous self-report study also found that anger was the reason most often given for interpersonal delinquency [27]. In addition, some previous studies reported that the perceivers' own emotional states influenced the recognition of others' emotional facial expressions (e.g., [28]). Taken together, the data suggest that delinquents might be projecting their own heightened angry emotions onto others when they misperceive others' negative, but not hostile, emotional states as anger.

Promising directions for further investigation include efforts to understand the developmental mechanisms for the impaired recognition of facial expressions in delinquents. One possible mechanism suggested by some studies involves a link between child maltreatment and subsequent delinquency [29,30]. Interestingly, consistent with our finding, Pollak et al. [31] found that maltreated children demonstrated impaired recognition of facial expressions of disgust, along with a misrecognition bias toward anger. Also, in keeping with previous findings for delinquents [10-12], studies have shown that maltreated children exhibited a bias toward attributing hostile intent to others [32,33]. The parallel between findings from these earlier studies and results of the present study suggest that impaired facial expression recognition in delinquents may be, at least in part, attributable to experiences of abuse during childhood.

Our results revealed differences in recognition accuracy in response to Caucasian and Japanese faces selected from standard stimulus sets [17,18]. This result suggests the possibility that cultural differences underpinned the finding that facial expressions depicting disgust were accurately recognized more frequently in response to Caucasian rather than Japanese models among both Japanese delinquent and control participants. However, we must note that the stimuli differed not only with regard to cultural dimensions but also with regard to some other properties. For example, whereas Caucasian stimuli included both young and middle-aged models, Japanese stimuli consisted solely of young models. Future studies might be necessary to confirm the cultural differences in expression recognition among delinquents.

Some potential limitations in the present study must be acknowledged. First, the mean IQ of the delinquents in this study was near the bottom of the normal range, raising the possibility that these delinquents showed impaired expression recognition partly because the task was too difficult for them. However, the MANCOVA revealed no significant influence of IQ on group differences. Furthermore, there was no significant group difference with respect to the recognition of fear, which is generally the most difficult to correctly recognize among emotions (cf., [34]). Consistent with this finding, previous studies investigating expression recognition in individuals with subnormal intelligence did not find specific impairment in the recognition of facial expressions of disgust or a misrecognition bias toward angry expressions [35]. These findings indicate that the impaired expression recognition in delinquents found in this study was attributable to a bias that was independent of intelligence level.

Second, the reaction times of responses were not recorded and analyzed in the present study. It is possible that different recognition performances derived from different cognitive processes, which could have been reflected in reaction times. Studies investigating reaction times will provide clues regarding the cognitive processes underlying expression recognition in delinquents.

Finally, confounding factors might have contributed to differences in expression recognition. For example, previous studies have shown that psychiatric disorders (e.g., schizophrenia [36]) and socio-economic status (e.g., economic disadvantages [37]) can influence expression recognition. In this study we were not able to access information on these issues due to the policies of the ministry that administrates the facilities. Furthermore, other studies have shown that the emotional states (e.g., state anxiety [28]) and personality traits (e.g., empathy [38]) of participants can affect expression recognition. These factors might have also influenced expression recognition in delinquents. Future research incorporating these factors should provide additional insights regarding expression recognition in delinquents.

## Conclusion

In summary, we found that the adolescent/young adult delinquents were impaired in their recognition of facial expressions of disgust. Their error patterns showed that they had a tendency to misrecognize facial expressions depicting disgust as anger. These results suggest that one factor underlying delinquency might be impairment in understanding emotions communicated by disgusted facial expressions, especially a tendency toward hostile interpretations.

## Competing interests

The authors declare that they have no competing interests.

## Authors' contributions

WS, NM, and MT designed this research. WS, SU, and NM collected the data. WS and SU analyzed data. WS, SU, and MT wrote the manuscript. All authors read and approved the final manuscript.
